# Factor XII-Driven Inflammatory Reactions with Implications for Anaphylaxis

**DOI:** 10.3389/fimmu.2017.01115

**Published:** 2017-09-15

**Authors:** Lysann Bender, Henri Weidmann, Stefan Rose-John, Thomas Renné, Andy T. Long

**Affiliations:** ^1^Institute of Clinical Chemistry and Laboratory Medicine, University Medical Center Hamburg-Eppendorf, Hamburg, Germany; ^2^Biochemical Institute, University of Kiel, Kiel, Germany; ^3^Clinical Chemistry, Department of Molecular Medicine and Surgery, L1:00 Karolinska Institutet and University Hospital, Stockholm, Sweden

**Keywords:** contact system, factor XII, kallikrein–kinin system, bradykinin, mast cells, heparin, polyP, anaphylaxis

## Abstract

Anaphylaxis is a life-threatening allergic reaction. It is triggered by the release of pro-inflammatory cytokines and mediators from mast cells and basophils in response to immunologic or non-immunologic mechanisms. Mediators that are released upon mast cell activation include the highly sulfated polysaccharide and inorganic polymer heparin and polyphosphate (polyP), respectively. Heparin and polyP supply a negative surface for factor XII (FXII) activation, a serine protease that drives contact system-mediated coagulation and inflammation. Activation of the FXII substrate plasma kallikrein leads to further activation of zymogen FXII and triggers the pro-inflammatory kallikrein–kinin system that results in the release of the mediator bradykinin (BK). The severity of anaphylaxis is correlated with the intensity of contact system activation, the magnitude of mast cell activation, and BK formation. The main inhibitor of the complement system, C1 esterase inhibitor, potently interferes with FXII activity, indicating a meaningful cross-link between complement and kallikrein–kinin systems. Deficiency in a functional C1 esterase inhibitor leads to a severe swelling disorder called hereditary angioedema (HAE). The significance of FXII in these disorders highlights the importance of studying how these processes are integrated and can be therapeutically targeted. In this review, we focus on how FXII integrates with inflammation and the complement system to cause anaphylaxis and HAE as well as highlight current diagnosis and treatments of BK-related diseases.

## Background of the Plasma Contact System

**Figure 1:**
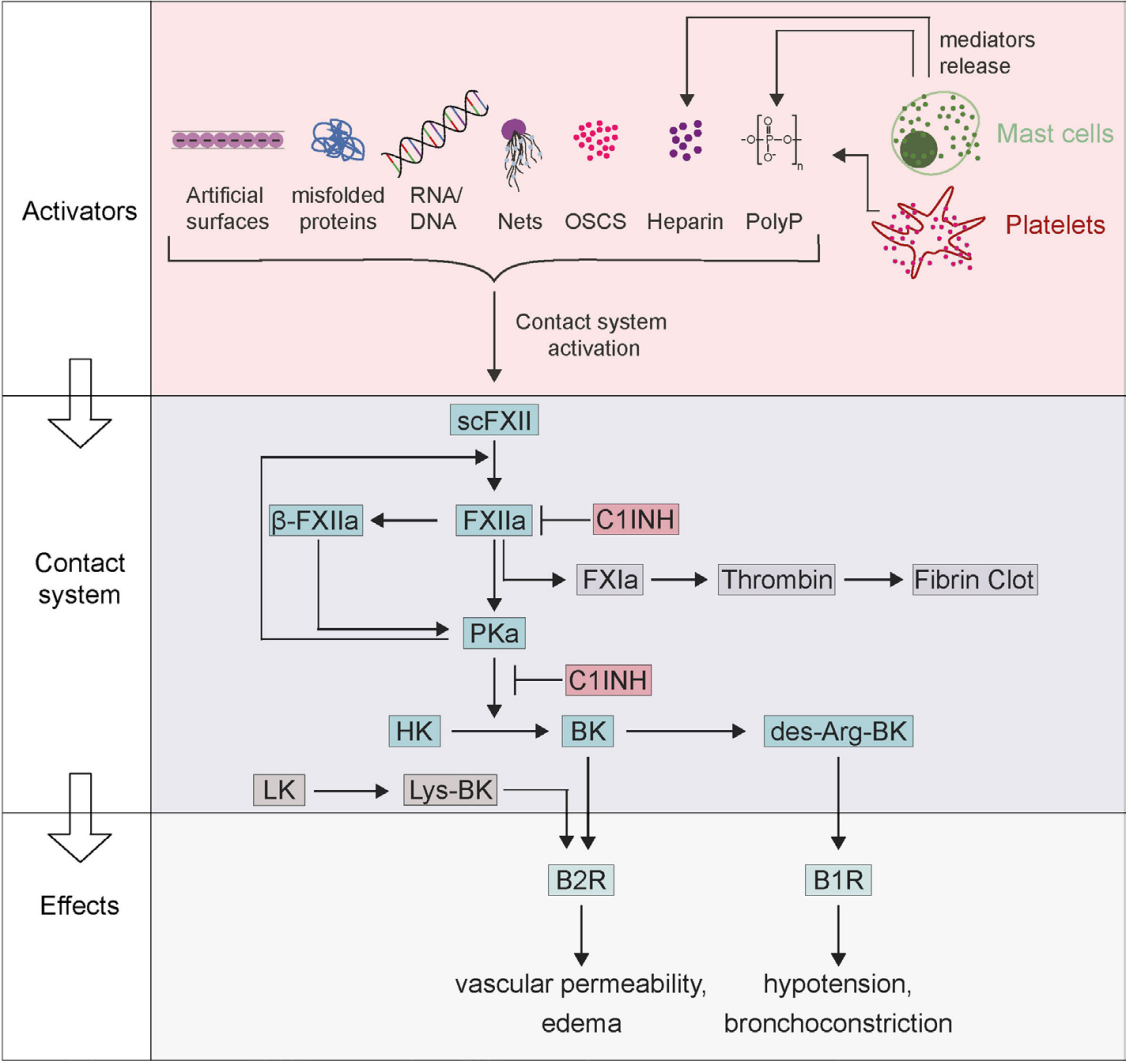
Factor XII (FXII)-driven contact system in activation of anaphylaxis. Zymogen scFXII becomes activated to FXIIa either by endogenous activators [misfolded proteins, RNA/DNA, neutrophil extracellular traps (NETs), polyP, oversulfated chondroitin sulfate-contaminated heparin (OSCS-heparin) and heparin] or by artificial surfaces. Anaphylaxis can activate mast cells with the release of their mediators (polyP and heparin), which also leads to FXIIa. FXIIa proceeds to activate prekallikrein, which reciprocally cleaves both FXIIa into β-FXIIa and high-molecular-weight kininogen (HK) to bradykinin (BK). BK binds receptor B2 (B2R) and triggers inflammation, edema, and symptoms of anaphylaxis. BK can be further proceeding to des-Arg-BK and mediates B1 receptor (B1R) activation resulting in hypotension and bronchoconstriction. The contact system can be inhibited by the C1INH that inhibits both FXIIa and plasma kallikrein.

The factor XII (FXII)-driven contact system is a network of proteases and inhibitors that integrates four major pathways: (1) the complement system, (2) the coagulation cascade, (3) the fibrinolytic system, and (4) the kallikrein–kinin system (1). The name “plasma contact system” comes from FXII being activated when it comes into “contact” with anionic surfaces, which leads to a conformational rearrangement resulting in the active protease factor XIIa (FXIIa). FXIIa initiates a series of downstream events that mediate the interface between of inflammation and coagulation (2, 3). FXIIa activates two serine proteinases, factor XI (FXI) and plasma prekallikrein (PK) that drive the coagulation and kallikrein–kinin systems, respectively. The non-enzymatic cofactor, high-molecular-weight kininogen (HK) is cleaved by activated plasma kallikrein (PKa) to release the pro-inflammatory oligopeptide bradykinin (BK) (4). Recent data have linked FXIIa-driven formation of BK and the downstream activation of the G-protein-coupled receptor B2 (B2R) potentially signaling to anaphylaxis and other immunologic disorders (Figure 1) (5, 6).

### Proteins and Molecules of the Contact System

#### Factor XII

Factor XII circulates in plasma as a zymogen with a concentration of 40 µg/ml (375 nM) (1). Coming into contact with anionic surfaces causes the zymogen form of FXII to undergo a conformational change in the presence of zinc ions. Conformational rearrangements induce auto-activation, which leads to small amounts of FXIIa (7). Due to activation of FXII zymogen, the single-chain polypeptide is converted into a two-chain molecule, composed of a heavy chain [353 amino acid (aa)] and a light chain (243 aa). The two chains stay connected with each other by a disulfide bond between Cys340 and Cys367 residues. The heavy chain is responsible for binding to anionic surfaces and, similar to HK heavy chain (8), links the zymogen to proteoglycans of cell surfaces (9). The catalytic domain is located within the C-terminal light chain of the protease. In humans, single-chain (sc)FXII has measurable, although much lower, proteolytic activity than complete FXIIa and its potential importance *in vivo* remains to be shown (10). FXIIa initiates the intrinsic coagulation cascade, which leads to the generation of thrombin and fibrin to produce clots in the blood (11). Furthermore, FXIIa converts PK to the active protease PKa, which reciprocally activates more FXII (7). In addition, PKa can initiate a further proteolysis of FXIIa into a ~30 kDa light chain fragment, termed β-FXIIa. The cleavage takes place at the peptide bond Arg353–Val354 and consequently, the active site released from the heavy chain and thus from surfaces. This small, soluble β-FXIIa variant retains its proteolytic activity toward PK, but not to FXI (Figure 1), offering an explanation for selective activation of the kallikrein–kinin pathway in the absence of coagulation (12).

#### Plasma Kallikrein

Prekallikrein has a plasma concentration of 35–50 µg/ml (580 nM) and exists as two different glycosylated forms with molecular weights of 85 and 88 kDa, respectively. Similar to FXII, a limited proteolysis activates zymogen PK and the active form is composed of a heavy chain (residues 1–371, 55 kDa) linked by a disulfide bond and a light chain (residues 378–619, 30 kDa). The heavy chain contains four apple domains and PK/PKa binding to HK is mediated by apple domains 1, 2, and 4 (13, 14). The PK light chain contains the peptidase domain with the substrates being HK, FXII, plasminogen, and urokinase-type plasminogen activator. Interestingly, the kallikrein–kinin system is linked to thrombosis, fibrinolysis, and the rennin–angiotensin system through the conversion of plasminogen to plasmin by PKa (7).

#### High-Molecular-Weight Kininogen

In humans, the non-enzymatic cofactor HK is generated from a single gene but undergoes alternative splicing to form high- (HK) and low-molecular (LK) weight kininogen. Murine HK contains two kininogen genes and both transcripts undergo alternative splicing which results in four kininogens. HK, but not LK, binds to cell surface glycosaminoglycans and the interaction is improved by zinc ions (15, 16). There is no detectable spontaneous HK activation due to HK protection from proteolytic cleavage by glycosaminoglycans binding. Therefore, cell surface presents a reservoir for BK production (7, 17).

### Activation of BK *via* the FXII-Driven Contact System

Bradykinin is a nonapeptide composed of the sequence Arg–Pro–Pro–Gly–Phe–Ser–Pro–Phe–Arg and functions as an inflammatory mediator. BK is the product of the kallikrein–kinin system following activation of FXII. FXIIa leads to proteolysis of PK, and the resulting PKa cleaves HK to generate BK (Figure 1). In contrast to PK, tissue kallikrein liberates kallidin (Lys-BK) from LK (18). Released BK binds with high-affinity (8–12 nM) to B2R. Upon binding of BK or kallidin, the activated B2R induces an increase of intracellular calcium ([Ca^2+^]_i_) that stimulates the endothelial nitric oxide synthase resulting in increased protein kinase G activity (19, 20). B2R signaling triggers vasodilatation, increase of vascular permeability, mobilization of arachidonic acid, and chemotaxis in granulocytes (21). BK increases vascular permeability *via* opening tight junctions of endothelial cells (22). B2R is constitutively expressed in multiple tissues such as endothelial cells, sensory fibers, smooth muscle cells, and epithelial cells, among others. Furthermore, expression of the B2R is enhanced by cytokines, cyclic adenosine monophosphate, estrogen, and glucocorticoids. Pathologic B2R activation contributes to various allergic, inflammatory, and infectious diseases such as sepsis, anaphylaxis, traumatic brain edema, rhinitis, capillary leak syndrome, or ischemia/reperfusion injuries (6, 23, 24). BK has a short half-life (<30 s) in plasma because it is quickly degraded by both plasma and endothelial peptidases. To overcome limitations in analyzing BK in patient samples elegant assays that measure BK-free HK (cleaved HK) have recently been developed (25). The angiotensin-converting enzyme (kinase 2), carboxypeptidases M and N (kininase 1), and the neutral endopeptidase (Neprilysin) process BK at two distinct sites (Pro7–Phe8 and Phe5–Ser6) leading to the inactive peptides BK1–7 and BK1–5 (26). Carboxypeptidase N removes the C-terminal BK arginine residue resulting in the metabolite des-Arg9-BK. This peptide stimulates the G-protein-coupled kinin B1 receptor (B1R) (27). Under normal physiological conditions, B1R is minimally expressed, but expression is rapidly upregulated in response to stimuli such as tissue injury or an increase in inflammation (20). Pharmacological inhibition of some mitogen-activated protein kinases and NF-κB interfere with B1R expression. Interestingly, all kallikrein–kinin system components are found within the central nervous system (CNS), and BK is formed and contributes to brain trauma and ischemia (28). Recently, a role for B1R in brain immune inflammation in a mouse model of Alzheimer’s disease was identified, possibly with microglial/macrophage involvement (29). Blocking B1R reduces brain infarction and edema formation in mice, while B2R deficiency had no effect on stroke outcome in mice (30). Furthermore, murine models indicate a role of FXIIa and BK in CNS autoimmunity, including multiple sclerosis (31) and pharmacologic interference with BK formation and/or signaling might ameliorate secondary brain injury (32).

## The Contact System Integrates with Activated Mast Cells, the Complement System and Mediates Anaphylaxis

Anaphylaxis is a multisystem syndrome of a rapid onset of symptoms and an immunologic response to allergens (33) that is predominantly driven by activated mast cells. Mast cells are found near blood vessels and areas susceptible to foreign antigens, such as tissue mucosa, and serve as multifunctional effector cells in the immune system (34). In most cases, the initiation of anaphylaxis is due to an antigen (allergen) that interacts with high-affinity receptors for immunoglobulin E (FcεRI), which are located on mast cells and basophils. Allergen-binding leads to intracellular signaling that results in the release of granules (35). These components, which are synthesized by mast cells and other immune cells such as macrophages or neutrophils, interact with circulating plasma proteins or tissue factors. Among the liberated compounds is histamine, which increases vascular permeability and vasodilation, leads to plasma leakage and reduced intravascular volume (36). This induces a drop in blood pressure that can lead to a lethal outcome.

### Mediators of the Mast Cells and Activators of FXII-Driven Contact System

Mast cells critically contribute to anaphylaxis. The link between mast cells and anaphylaxis was established once it was discovered that mast cells were abundant in protein and mediators such as tryptase, chymase, and other cytokines, as well as newly synthesized lipid-derived molecules such as prostaglandins, platelet-activating factor (PAF), cytokine tumor necrosis factor α, and leukotrienes (37). These mediators play an important role in the development of anaphylaxis; however, the mechanisms of inducing anaphylaxis vary widely (6). For instance, PAF activates inflammatory and thrombotic pathways by causing platelet activation and liberates vasoactive substances, resulting in increased endothelial permeability. Uncontrolled PAF activities can result in sepsis, shock and are important in disseminated intravascular coagulation (38, 39). Prostaglandins lead to smooth muscle relaxation and act as vasodilators. Interestingly, they can also inhibit platelet adherence. Levels of urinary prostaglandin D2 correlate with severity of anaphylaxis (40) and leukotriene production accompanies histamine and prostaglandin production. Their release triggers smooth muscle contractions and vasodilation, leading to bronchoconstriction and hypotension. Cysteinyl leukotrienes are termed slow-reacting substance of anaphylaxis and are up to 1,000-fold more potent than histamine but have a slower onset and long-lasting activities (41–43). The overlap of these pathways leads to synergistic pathologic effects that also result in activation of complement and contact system pathways, highlighting the importance of developing effective therapeutics for this potentially lethal condition. In this section, some of the main mediators that induce inflammation and/or coagulation through contact system-mediated pathways will be discussed in greater detail.

#### Histamine

Released histamine causes increased angioedema, anaphylaxis, or chronic spontaneous urticaria and is also involved in allergic responses. Histidine decarboxylase is the only enzyme capable of producing histamine (44). Upon mast cell release, histamine promotes recruitment of T_H_2 helper cells and dendritic cells along with antigen presentation (35). Mast cell secretory granules also contain heparin and proteoglycans, which are heavily negatively charged, in contrast to histamine, which is positively charged. Both components can interact within granules and upon mast cell activation, heparin proteoglycans and histamine are released with similar kinetics (45). Furthermore, histamine and heparin have been shown to interact in purified systems (34, 46), but there is no evidence for a physiologically relevant interaction *in vivo*. In urticaria patients, the occurrence of angioedema was reduced with antihistamine therapy (47). In addition, there were no increased plasma BK levels in four patients with an acute histamine-sensitive angioedema (48) arguing that BK and histamine have the capacity for inducing edema by independent pathways. For angioedema with unknown derivation (idiopathic angioedema) and for hereditary angioedema (HAE), histamine receptor antagonists are clinically applied, but approximately one in six patients exhibiting idiopathic angioedema do not respond to antihistamine treatments (49). This suggests that other mediators are involved in the trigger and the outcome of hereditary forms of angioedema.

#### Serotonin

Serotonin, a biogenic amine, is a mast cell granule constituent. However, confocal microscopy revealed that distinct mast cell granules contain both histamine and serotonin (50, 51). In absence of endogenous histamine, serotonin is increased in immune cells including mast cells (44). One explanation could be that mast cells can selectively release serotonin without releasing histamine (52) *via* high-affinity serotonin-binding proteins used to sequester serotonin from secretory vesicles (53). Serotonin functions as a regulator of immune and inflammatory responses and is partially mediated through direct interactions with macrophages (54).

#### Heparin

Another major component of mast cell granules is heparin, which is released following IgE/antigen activation (5). Heparin-driven FXII contact activation triggers the kallikrein–kinin system, releases BK to stimulate B2R in human plasma and leads to edema *in vivo* (55). Heparin levels are elevated in patients with anaphylaxis while PK and HK plasma levels are low in anaphylaxis, indicating that the contact system is indeed activated. In contrast to other contact system activators, mast cell heparin does not activate the coagulation pathway, possibly because heparin binds to antithrombin III, thereby increasing its inhibitory activity toward thrombin (6).

In 2007, heparin contaminated with synthetic oversulfated chondroitin sulfate-contaminated heparin (OSCS-heparin) was accidentally given to patients in the United States and Germany. This commercially available contaminated heparin resulted in adverse clinical events in the heparin therapy for hundreds of individuals (56). Within several minutes of intravenous infusion of contaminated heparin, there was a drastic reaction in patients causing edema, hypotension, swelling of the larynx and other related symptoms including death (56). The OSCS-contaminated heparin potently activates FXII *via* the kallikrein–kinin system through BK formation in human plasma (57), demonstrating the importance of understanding the mechanisms that induce BK in patients.

#### Polyphosphate

Polyphosphate (polyP) is a polymer of linear linked phosphate units *via* energy-rich phosphoanhydrous bonds. PolyP is pro-inflammatory and procoagulant and is found in secretory granules of platelets, basophils, and mast cells that resemble acidocalcisomes in prokaryotes (51, 58). Mast cell activation leads to a release of polyP that activates the FXII-driven contact system (51) while FXII- or B2R-deficient mice do not exhibit activated mast cell-induced edema and hypotension (5).

Polyphosphate was first found in prokaryotes and is involved in metabolism, structural behavior and stress responses. The polymer can be from a few up to thousands of residues long (11). In artificial systems, dissolved long-chain polyP (>500 residues) activates FXII more potently than short-chain polyP (<100 residues); however, these long-chain polymers have low solubility under physiological conditions (59). The hypothesis that size determines the activity of polyP for activating FXII has been challenged by the fact that polyP form calcium-rich nanoparticles *in vivo*. Independent of the size of the individual, polyP monomer polyP, packed into particles potently activates FXII (60). PolyP is unstable in plasma (61) and technology to specifically analyze the polymer has been developed (62). Recently, intravital microscopy visualized release of polyP nanoparticles from platelet dense granules. PolyP nanoparticles accumulate on the procoagulant platelet surface *in vivo*. The polyP particles are retained on the platelet surface where they potently initiate FXII contact activation (63, 64). FXII activation by exposed procoagulant polyP offers a rationale for the critical role of FXIIa in mediating platelet driven coagulation/clot formation that is well established since decades the field (65–70). In addition to polyP particles, small amounts of short-chain soluble polymers are released into the supernatant from activated platelets (71) and activate an array of procoagulant mechanisms (72). The role of these FXII-independent mechanisms, however, remains enigmatic *in vivo*. PolyP colocalizes with serotonin and calcium in the acidic secretory granules of mast cells (51). Taken together, polyP in mast cells is released in a mechanism similar to that of platelets. These data suggest that the release of heparin coupled with polyP inhibits the procoagulant properties of polyP while retaining the pro-inflammatory capability.

### Contact System Cross Talk with the Complement System

An important component of the immune response is the complement system, which is composed of soluble proteins circulating as precursors in the plasma. There are three distinct pathways that can activate the complement system: (1) the classical pathway, (2) the lectin pathway, and (3) the alternative pathway. The classical pathway is activated *via* binding of C1q to antibodies complexed with antigens. In some cases, the interaction of C1q with certain pathogens can lead to a direct surface binding without the presence of antibodies. The C1 complex contains C1q, which is further bound to two molecules each of the zymogens C1r and C1s (73). The mannose-binding lectin (MBL) pathway is initiated when mannan-binding lectin-associated serine proteases (MASP-1 and MASP-2) bind and are activated *via* MBL, ficolins or collectins to carbohydrates on the bacterial cell wall (12). The alternative pathway is initiated *via* spontaneous activation of C3b that leads to binding on the pathogenic surface. The activation of all three pathways is driven by a series of limited-proteolysis reactions that convert the proenzymes to an active enzymes (74) culminating in generation of C3 convertase (73). The convertase cleaves C3 to C3a and C3b and can generate more C3 molecules to amplify production of C3b. C3b is involved in the production of C5 convertase, which functions as an opsonization marker for bacteria to be phagocytosed by macrophages and neutrophils (75). The cleavage of C5 by C5 convertase yields C5a and C5b in a similar fashion as C3. C3a and C5a, known as anaphylatoxins, are pleiotropic inflammatory mediators and proteolytically released from C3 and C5 (74). In host defense responses, the membrane attack complex (MAC) is produced by C5b-mediated formation of C5b-9 complex. This MAC induces lysis of pathogens or cells *via* incorporation into the cell membranes (12).

The complement system has the capacity to trigger anaphylactic shock, mainly *via* C3a and C5a activity. These anaphylatoxins induce degranulation of mast cells, which leads to the release of histamine. In addition, they also increase vascular permeability and induce contraction of smooth muscle cells (76, 77). Complement activation was found to trigger anaphylactic shock in mice exposed to peanut extract through C3 activation. In accordance with this finding, the authors showed that mice deficient in C3 or its receptor C3aR had almost no response to the peanut extract (78).

**Figure 2:**
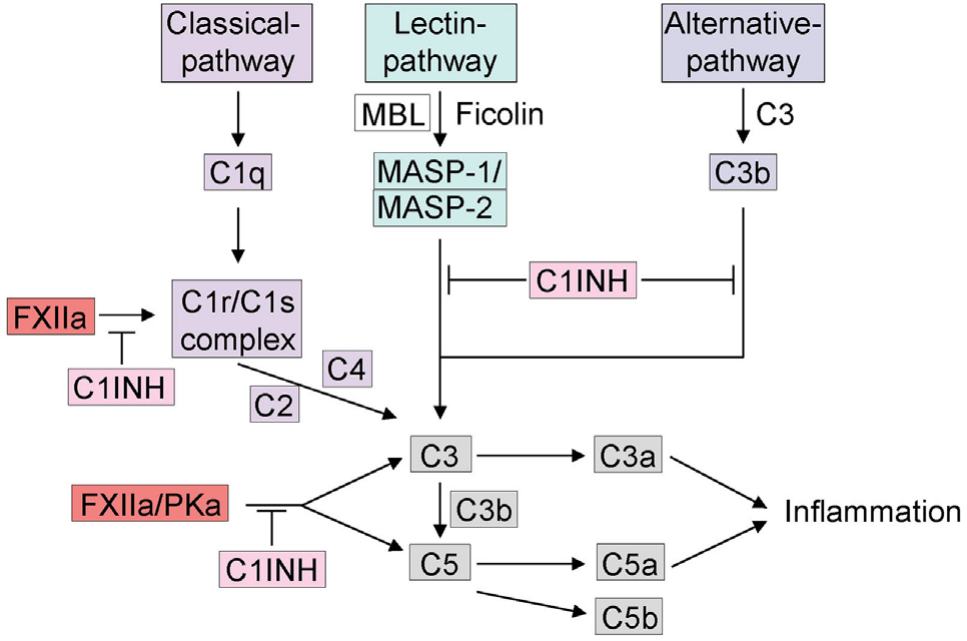
Cross talk between the kallikrein–kinin system and the complement system. The complement system can be activated by three different pathways: (1) C1q initiates the activation of the classical pathway, (2) the mannose-binding lectin (MBL) or ficolins trigger the lectin pathway for glycosylation on the surface of pathogens. Activation of either the classical pathway or MBL generates C3 convertase. (3) If C3 is spontaneously hydrolyzed, the alternative pathway is activated and generates activated C3b. C3 and C5 release C3a and C5a, which can trigger inflammation. The activation of the complement system *via* the kallikrein–kinin system is indicated, mainly FXIIa and plasma kallikrein are involved in activation of C3 and C5. FXIIa can trigger the C1r/C1s complex. C1INH inhibits the complement system in all three different pathways and furthermore *via* the inhibition of the kallikrein–kinin system.

There is extensive cross talk between the complement and contact systems at several levels (Figure 2). They share the major endogenous inhibitor, C1INH that inhibits the initial step of both cascades. While C1INH inhibits FXIIa activation of the contact system, all three-activation pathways of the complement are also inhibited by distinct mechanisms. The classical pathway is inhibited by C1INH-mediated inactivation of C1r and C1s (79), two subunits of the C1 complex that is also known to be activated by FXIIa (Figure 2) (80, 81). By covalent binding to MASP-1 and MASP-2, C1INH also inhibits the lectin pathway (82). Finally, the alternative pathway is inhibited by reversible binding of C1INH to C3b (83). Interestingly, *in vitro* activation of FXII by OSCS activates C3 and C5 in human plasma in addition to the kallikrein–kinin system. In FXII-deficient plasma, activation was abrogated with no effect on normal complement activation, an effect that was rescued by addition of purified FXII to FXII-deficient plasma (57). There are multiple inhibitors of the complement system that are expressed on cell surfaces. The inhibitory effect of antithrombin, however, is much enhanced by glycosaminoglycans, such as heparin and heparan sulfate (84, 85). Further interactions between the complement and the kallikrein–kinin system have been discovered. For example, PKa has been found to trigger the generation of C3a fragments in humans (86) and C5a due to limited proteolysis of C5 in rabbits (87). While this last reaction was confirmed using anti-PK IgG or soybean trypsin inhibitor, this result has yet to be confirmed in humans. The cross talk between complement and the contact system has become increasingly more relevant because many types of molecules are produced that play an important role in pathologies such as angioedema (12) and anaphylaxis.

## BK in Anaphylaxis and Inflammatory Diseases

### Anaphylaxis

Anaphylaxis can result from serious allergic reactions and immunologic response to allergens and may lead to life-threatening swelling episodes (33). Its onset is in the range of a few minutes if the allergen entered *via* the circulatory system to a couple of hours if the allergen was ingested. Depending on the type of the response, symptoms of anaphylaxis include hypotension, vascular leakage, or even cardiac arrhythmia and bronchial constriction in severe cases (6). There are common triggers for anaphylactic reactions such as food, medications or insect venom with 1–15% of the population being susceptible to anaphylaxis (88).

Recent work from our group has shown that this increased vascular permeability was mediated by heparin-initiated BK formation in mice (5). In this study, it was shown that targeting FXII or B2R abrogated heparin-mediated leukocyte adhesion to the endothelium and inhibited mast cell-triggered hypotension. Ablation of FXII or B2R protected against mast cell-mediated leakage in response to allergens and heparin-induced edema. Furthermore, our group has also demonstrated that deficiency or targeted inhibition of FXII, PK, HK, B2R, but not B1R, resulted in a protective effect against anaphylaxis in an allergen/IgE mice model. In *F12^−/−^* mice, this protective effect could be abolished by restoration of plasma FXII levels, confirming the involvement of the contact system in this model of anaphylaxis. Analysis of human plasma from anaphylactic patients revealed activation of the contact system. The degree of anaphylaxis associated with levels of mast cell degranulation, heparin levels in the plasma, the amount of contact activation, and subsequent BK formation (6).

Abnormal blood coagulation as a result of IgE-triggered hypersensitivity has been known for years. Activated partial thromboplastin time, a measure of FXIIa-driven coagulation is delayed in patients with anaphylaxis and anaphylactic shock (89, 90). In contrast, the prothrombin time, which utilizes the FXII-independent extrinsic pathway of coagulation, remains unchanged in patients with allergen-mediated anaphylaxis, suggesting that they mediate their effect only *via* the intrinsic coagulation pathway (11). The plasma of IgE/Ag-challenged mice does not clot due to a heparin concentration of >4 μg/ml, which is sufficient for initiation of BK formation (5). Therefore, minute amounts of heparin may produce BK on the mast cells surface.

### Hereditary Angioedema

Dysregulation of the contact system leads to HAE, an autosomal dominant disorder that results in recurrent episodes of angioedema of the skin or tissue mucosa. Before the use of prophylactic drugs, laryngeal edema and upper airway obstruction were lethal in up to one-third of patients (91). It is unknown how prevalent HAE is across the world but current estimates propose as many as 1/10,000–1/150,000 individuals in Europe (92). HAE is caused by either reduced C1INH levels (HAE type I) (93), a defective C1INH protein (HAE type II) (94), or hyperactive FXII (HAE type III). In HAE type III patients, C1INH functions normally and circulates at a normal concentration in plasma. However, a single point mutation in FXII (position 309) leads to enhanced FXIIa activity by a mechanism that recently has been unraveled (95). A defective FXII glycosylation at that single site (Thr309 that is mutated to Arg or Lys) is the underlying cause of excessive FXII activation in HAE type III, suggesting that HAE type III is a disease model for gain of function FXII contact activation (96). Edema in HAE type III is not associated with thrombosis (96), supporting a role of mast cell heparin in activating mutant FXII similar to anaphylactic reactions (6). C1INH deficiency increases the ability of FXIIa to convert PPK to PKa (97), since C1INH inhibits over 90% of plasma FXIIa (94). In murine models, cross-breeding C1INH-null with B2R-null mice completely rescues the leakage phenotype, confirming that BK triggers edema formation (98). During acute swelling attacks, C1INH infusions, B2R antagonists, and PK inhibitors have all been shown to effectively block generation of BK (20).

### Inflammatory Diseases

Vasodilation and vascular permeability are two processes are involved in many inflammatory diseases (48), leading to local swelling attacks of the dermis and submucosa (99). As an important regulator of those processes, the contact system has been studied in several inflammatory diseases. In rheumatoid arthritis and irritable bowel diseases, for example, high levels of PKa and BK have been observed. Furthermore, it was shown in rodent rheumatoid arthritis models that inhibition of the contact system interferes with arthritis. Moreover, HK deficiency in rats resulted in less acute and chronic arthritis (100). The precise role of the kinin receptors in rheumatoid arthritis has remained a matter of discussions. While B2R receptor deficiency did not affect arthritis in a mouse model of anti-collagen antibody-induced arthritis, combined deficiency of B2R and B1R attenuated arthritis (101). In support of these observations, there are similar findings in irritable bowel disease. Patients with ulcerative colitis (UC) showed decreased plasma levels of PKa and HK, which indicated proteolysis of these precursors and therefore contact system activation (102). Both kinin receptors are expressed in UC patients in intestinal epithelial cells. During active UC, however, B1R is significantly upregulated and seems to be the main receptor by which BK exerts its deleterious effect in UC (18). Interestingly, in a murine dextran sulfate induced colitis model C3, deficiency conferred protection from disease development indicating a role for the complement system in the disease (103). In the same study, the authors showed that treatment with C1INH would also reduce the severity of the disease in WT mice. A rat enterocolitis model confirmed the clinical observation of decreased plasma PKa and HK and intestinal inflammation could be reduced by treatment with BK antagonists or HK deficiency in a PG-PS model. There are many different animal models that display intestinal inflammation and contact system inflammation but use different triggering agents (56, 104, 105), suggesting that the contact system is an integral part of the process. Taken together, these data indicate that contact activation can be detected in most inflammatory diseases and is mostly mediated through BK production and its receptors.

### Diagnostics Related to the Kallikrein–Kinin System

Diagnostics for anaphylaxis are well described in Montanez et al. (106). In the case of BK-related anaphylaxis, there are some more *in vitro* assays available. But measuring the concentration of BK is very challenging, due to rapid degradation of BK and des-Arg9-BK (27 ± 10 and 643 ± 436 s, respectively) (107). Therefore, a number of enzymatic assays have been designed to circumnavigate this issue by measuring more stable BK-related products such as cleaved HK levels (108, 109). Other assays focus on C1INH inhibitory capacities by measuring free C1s activity (110) and C1INH-protease complexes levels (111).

#### Amidase Activity Assay

This assay measures the activity of free, active C1s amidase (e.g., not bound to C1INH) by the kinetic or endpoint colorimetric assay, using the substrate H-d–Pro–Phe–Arg–pNA (110). It was demonstrated that spontaneous amidase activity was increased in plasma from patients with BK-dependent disorders compared to plasma from normal patients. They confirmed increased BK production by detecting HK cleavage *via* Western blot, which also correlated with increased kininogenase activity (112).

#### Cleaved Kininogen Assay

A direct indicator of BK release is cleaved HK (25, 113). The reconstitution of liver-synthesized, novel protein is slow. Due to the slow recovery of plasma HK levels, the observed distribution of HK and HK degradation products gives a robust readout of the *in vivo* BK production and allows for the detection of active angioedema (114). The cleaved kininogen assay could be used in injury cases where the role of contact system is developing, such as in transfusion-related acute lung injury and other detrimental blood reactions (115).

### Treatments with Drugs against BK Formation

The standard treatment for anaphylaxis is adrenaline, but since mast cell and contact system activation correlate with the severity of the response (116), other drugs inhibiting BK formation could be also considered. For example, specific inhibitors of the kallikrein–kinin system have been shown to be effective at preventing BK-mediated HAE attacks. Some severe side effects can exist, such as a hypersensitivity to the drug that can induce anaphylaxis. The variations in physiological responses demonstrate the need for detailed mechanistic studies of therapeutics that target the contact system. Some of the current therapeutics in clinical trials will be discussed in more detail.

#### Icatibant

Icatibant (Firazyr^®^; Shire) is a synthetic decapeptide containing five non-proteinogenic amino acids (H–d-Arg–Arg–Pro–Hyp–Gly–Thi–Ser-d–Tic–Oic–Arg–OH) which resembles the BK-peptide and selectively blocks B2R. In contrast to BK, icatibant has a relatively long half-life (1–2 h) (117). Several *in vitro* and *in vivo* pharmacological assays showed that icatibant binds with a high-affinity to B2R in guinea pig models (118). Furthermore, the anaphylaxis associated BK-induced bronchoconstriction in guinea pig models was inhibited with icatibant (119). Consistent with animal model data, clinical trials showed the efficacy of icatibant and a strong decrease of HAE attacks in treated patients (114, 120). Some side effects were observed in 90% of the patients treated with icatibant, such as temporally local pain, swelling, and erythema at the injection site.

#### Ecallantide

Ecallantide (Kalbitor^®^; Dyax, USA) is a potent recombinant protein modeled after the human tissue factor pathway inhibitor Kunitz 1 domain that inhibits PK (121). To test the efficacy and safety in acute attacks, there were two double-blind, placebo-controlled studies performed in 160 patients with HAE. The results of these studies were comparable but the measurement of the patients’ reported outcomes was different (122, 123). A known risk of ecallantide treatment for acute HAE attacks is hypersensitivity and subsequent anaphylaxis. The clinical relevance and post-marketing surveillance are required to determine the therapeutic and clinical value (121).

#### C1INH

Some drugs are available to cover the impropriate function of C1INH or C1INH deficiency. These are plasma-derived (pd)C1INHs [Berinert^®^ (CSL Behring), Cetor^®^ (Sanquin), and Cinryze^®^ (ViroPharma)] or recombinant human (rh)C1INH (Ruconest^®^ in Europe, Rhucin^®^ in the USA, Pharming Group NV). The pdC1INHs prepared and pasteurized from fractionated plasma. rhC1INH is expressed in the mammary gland of transgenic rabbits. Interestingly, both synthetic proteins produced C1INHs differ in their glycosylation pattern. rhC1INH contains less glycosylation than pdC1INH due to its production in a heterologous system. Because of the differences in glycosylation patterns, the rhC1INH can be cleared within 3 h from the circulation, in comparison to pdC1INH, which takes more than 24 h. It is important to know if patients have a rabbit allergy since this could induce anaphylaxis upon treatment with rhC1INH (121). To confirm the safety of these products, more long-term data are necessary.

#### Avoralstat

Avoralstat (BCX4161) is developed by BioCryst Pharmaceuticals Ltd. (Durham, NC, USA) and is a small molecule kallikrein inhibitor of oral administration. It is an effective and specific inhibitor of PK, as indicated in preclinical studies. One promising study in phase IIa was performed with statistically significant mean attack reduction for HAE type I and type II (124).

#### DX-2930

DX-2930 is a recombinant human monoclonal antibody against PK produced by Dyax Corp (Burlington, MA, USA) that was developed using phage display. DX-2930 acts as a long-acting inhibitor and could be used to prevent HAE attacks (124).

#### Anti-FXIIa Antibody (3F7)

3F7 is a recombinant, fully humanized antibody (3F7) which neutralizes FXIIa by blocking the protease activity of the catalytic domain (125). 3F7 blocks the intrinsic clotting cascade in human plasma and thrombosis formation in mouse models. Consistent with the selective role of FXII in thrombosis but not in hemostatic mechanisms, 3F7 thromboprotection is similar to that of heparin but there is no change in bleeding. 3F7 interferes with FXII activation in response to an array of contact activators including polyP and heparin (126). In humanized mouse models of HAE type III, 3F7 inhibits FXIIa and as a consequence prevents edema in animal models. Supporting a potential use of 3F7 to treat anaphylaxis and HAE, the addition of the antibody abolished BK formation in patients’ plasma of HAE type III (96).

## Conclusion

The FXII-driven contact system plays a role in anaphylaxis and angioedema *via* its ability to increase inflammation and vessel permeability. During the onset of these pathologies, the mast cell activation releases pro-inflammatory mediators including polyP and heparin that can activate the contact system. This contact system activation triggers the kallikrein–kinin system and the complement pathways that intertwine at many levels, for example frequently used control mechanisms, cross-activation, and commonly used binding proteins. The abnormal production of BK leads to HAE and also plays a role in anaphylaxis that both can lead to acute, life-threatening attacks of edema. Therefore, it is of interest to study the common pathways between these pathologies. There are several novel drugs emerging to interfere with contact system activation and possibly other pathologies involving HK, BK, and C1INH. Further clinical studies of the contact system are required to better understand the connection between the contact system and inflammatory-related pathologies like HAE and anaphylaxis.

## Author Contributions

All authors have made significant intellectual contributions of the review. LB, HW and ATL drafted the original manuscript. SRJ and TR critically analyzed and gave suggestions for the concept and revision that improved content of the text and figures. All authors approved the final version of the manuscript.

## Conflict of Interest Statement

The authors declare that the research was conducted in the absence of any commercial or financial relationships that could be construed as a potential conflict of interest.
